# From Skin to Brain: Key Genetic Mediators Associating Cutaneous Inflammation and Neurodegenerative Diseases

**DOI:** 10.3390/genes16121463

**Published:** 2025-12-08

**Authors:** Vasiliki-Sofia Grech, Kleomenis Lotsaris, Vassiliki Kefala, Efstathios Rallis

**Affiliations:** 1 Department of Biomedical Sciences, School of Health and Care Sciences, University of West Attica, GR-12243 Athens, Greece; valiakef@uniwa.gr (V.K.); erallis@uniwa.gr (E.R.); 2 Department of Psychiatry, General Hospital of Athens: “Evaggelismos”, GR-10676 Athens, Greece; psych.kleolots@gmail.com

**Keywords:** psoriasis, rosacea, atopic dermatitis, bullous pemphigoid, Alzheimer’s disease, Parkinson’s disease, dementia, genetics, GWAS, skin–brain axis

## Abstract

Chronic inflammatory skin diseases and neurodegenerative disorders share overlapping genetic, immunologic, and metabolic pathways that may predispose individuals to cognitive decline. This review synthesizes current human genomic, transcriptomic, and bioinformatic evidence linking psoriasis, rosacea, atopic dermatitis, and bullous pemphigoid with Alzheimer’s and Parkinson’s disease. Literature from PubMed, IEEE Xplore, and Google Scholar was examined, prioritizing studies integrating genomic, transcriptomic, and proteomic analyses. Among inflammatory dermatoses, psoriasis exhibits the strongest overlap with dementia genetics, with shared susceptibility loci including *APOE*, *IL12B*, and *HLA-DRB5*, and transcriptional regulators such as *ZNF384* that converge on IL-17/TNF signaling. Rare-variant and pleiotropy analyses further implicate *SETD1A* and *BC070367* in psoriasis–Parkinson’s comorbidity. Rosacea demonstrates upregulation of neurodegeneration-related proteins SNCA, GSK3B, and HSPA8, together with shared regulatory hubs (*PPARG*, *STAT4*, *RORA*) driving NF-κB/IL-17/TNF-dependent inflammation. In atopic dermatitis, rare *FLG* variants interacting with *BACE1* suggest a mechanistic bridge between barrier dysfunction and amyloidogenic processing. Bullous pemphigoid reveals an HLA-DQB1*03:01-mediated immunogenetic link hypothesis and cross-reactive autoantibodies targeting BP180 (collagen XVII) and BP230, highlighting an autoimmune route of neurocutaneous interaction. Other inflammatory and neurodegenerative diseases with currently weak or limited genetic evidence are also discussed, as they may represent emerging biological pathways or potential therapeutic targets within the skin–brain connection in the future. The aim of this work is to help clarify these genetic links and to advocate for the routine cognitive assessment of affected patients, enabling early detection, improved long-term quality of life, and the potential for timely therapeutic intervention.

## 1. Introduction

The concept of the skin–brain axis describes a complex bidirectional network of interactions between the integumentary and central nervous systems. Their relationship originates during embryogenesis, as both organs derive from the ectoderm and develop in parallel during gastrulation. Such a shared developmental link has long suggested a profound biological and functional interconnection between the two systems. Following gastrulation, the fate of ectodermal cells to differentiate into neural tissue or epidermal epithelium, is determined by a tightly regulated interplay of molecular cues, primarily involving Wnt, fibroblast growth factor (FGF), and bone morphogenetic protein (BMP) signaling pathways. Active FGF signaling promotes neural induction, whereas its inhibition, accompanied by enhanced BMP activity, drives epidermal differentiation. Wnt signaling modulates this balance by restricting the ectoderm’s responsiveness to FGFs, ensuring proper regionalization between neural and non-neural ectoderm [1]. These developmental mechanisms not only define the early divergence of the nervous system and skin but also establish the molecular basis for their lifelong physiological cross-talk. The skin and brain communicate through immune, endocrine, vascular, and neural pathways that collectively maintain systemic homeostasis and influence the pathogenesis of diverse disorders [2,3]. Hence, this shared embryological and molecular framework provides a conceptual foundation for exploring the link between cutaneous and neurological diseases, highlighting an emerging interdisciplinary field that bridges dermatology, neurobiology, and genomic medicine.

Building upon this shared developmental and structural foundation, growing evidence indicates that skin inflammation can induce systemic effects that extend well beyond the cutaneous environment, while perturbations originating in the central nervous system (CNS) can, in turn, influence skin homeostasis [4,5]. For instance, based on the frequency of depressive symptoms experienced by patients with chronic wounds, Hadian et al. [5] proposed that chronic inflammatory skin states act as reservoirs of systemic inflammatory mediators that propagate neuroinflammation and contribute to cognitive and neuropsychiatric disturbances. Sustained activation of cutaneous immune pathways appears to promote the release of pro-inflammatory cytokines and danger signals into the circulation, leading to blood–brain barrier (BBB) dysfunction, activation of central immune responses, and altered neurotransmission [5]. Conversely, disturbances within the CNS, including chronic stress, major depressive disorder, and other neuropsychiatric conditions, can feed back to the skin via neuroendocrine pathways, particularly through the hypothalamic–pituitary–adrenal (HPA) axis, thereby exacerbating immune dysregulation and inflammatory dermatoses [2].

Furthermore, a growing body of epidemiological evidence provides robust support for the association between chronic inflammatory dermatoses and cognitive decline. Large population-based cohorts have consistently demonstrated that individuals with psoriasis, atopic dermatitis, and other eczematous conditions exhibit higher rates of mild cognitive impairment (MCI) and dementia compared with unaffected controls, independent of confounding variables such as age, sex, education, and cardiovascular risk factors [6,7,8]. For what is more, in psoriasis, this association appears to follow a disease–severity gradient, suggesting a potential dose–response relationship [8]. The relationship is particularly pronounced in older adults, where impaired epidermal barrier function enhances systemic cytokine release and perpetuates a state of chronic low-grade inflammation, often referred to as “inflammaging” [8,9].

This chronic systemic inflammation, commonly seen in the elderly, is characterized by elevated circulating cytokines such as IL-6, TNF-α, and IL-1β which are implicated in both neuroinflammatory processes and neurodegenerative disorders. These cytokines, produced by adipose tissue, endothelial cells, and activated immune populations, induce oxidative stress and mitochondrial dysfunction, amplifying the generation of reactive oxygen species (ROS) and sustaining local neuroinflammation. Concurrently, cytokine-driven endothelial activation upregulates adhesion molecules including ICAM-1 and VCAM-1, facilitating leukocyte adhesion and migration into neural tissue. Within the CNS, these immune mediators activate microglia and astrocytes, leading to persistent production of inflammatory factors that impair neuronal survival and synaptic integrity. This self-perpetuating inflammatory cascade, initiated by peripheral cytokines, ultimately contributes to neurodegenerative pathology through neuronal injury, loss of homeostasis, and accumulation of pathogenic proteins such as amyloid-β and tau [10].

Epigenetic modifications act as a dynamic molecular interface connecting chronic cutaneous inflammation with neurodegenerative processes by regulating gene expression without altering the underlying DNA sequence. In inflammatory skin diseases such as psoriasis and atopic dermatitis, DNA methylation is a key determinant of immune activation and epidermal barrier integrity. Genome-wide methylation studies reveal aberrant CpG methylation in promoters of cytokine-related genes (*TNF-α*, *IL6*, *IL17*, *IL13*, *IL4R*), leading to their overexpression and sustaining Th1/Th17- and Th2-driven inflammation. Likewise, alterations in histone acetylation and methylation (H3K9ac, H3K27ac, H3K27me3) modify chromatin accessibility, enabling transcription of inflammatory mediators while repressing genes involved in keratinocyte differentiation and skin homeostasis. MicroRNAs such as *miR-21*, *miR-146a*, and *miR-155* further amplify cytokine signaling and epidermal hyperplasia [11].

Parallel epigenetic disruptions occur in neurodegenerative diseases. In Alzheimer’s and Parkinson’s disease, dysregulated DNA methylation affects neuronal genes involved in synaptic plasticity, oxidative stress, and protein aggregation—including *APP*, *SNCA*, and *MAPT*—while aberrant histone deacetylation (e.g., loss of H3K27ac, H4K16ac) silences neuroprotective promoters such as *BDNF* and *CREB*. Non-coding RNAs modulate microglial activation and apoptotic signaling, linking chromatin remodeling to neuroinflammation and neuronal loss [12].

Collectively, findings from dermatologic and neurologic epigenome studies indicate that DNA methylation, histone modification, and non-coding RNA regulation form a shared epigenetic framework through which chronic inflammation, oxidative stress, and metabolic imbalance influence both cutaneous immune dysregulation and neurodegenerative pathology—providing a unifying chromatin-level mechanism that bridges skin inflammation and brain decline.

All of these reciprocal mechanisms create a biological bridge between cutaneous inflammation and cognition, with cognition being a set of higher mental processes that include perception, memory, attention, learning, and decision-making. These abilities collectively sustain autonomy and enrich quality of life. When these domains become dysregulated, the resulting cognitive impairment may manifest as subtle, transient lapses or progress insidiously toward irreversible decline. At its most severe stage lies dementia, with Alzheimer’s disease (AD) standing as its most prevalent form worldwide, though other neurodegenerative disorders, such as Parkinson’s disease (PD), likewise erode the very faculties that define human thought and selfhood [13,14].

Skin inflammatory diseases represent easily assessable factors during clinical examination, offering valuable and non-invasive information for patient evaluation. Similarly, sex constitutes an even more accessible variable in this context, with recent machine learning (ML) approaches increasingly associating sex with the prediction of neurodegenerative diseases and highlighting its role as a biological determinant of disease onset and progression. In an explainable ML study on PD, Angelini et al. [15] applied the CatBoost algorithm with SHAP analysis to data from the Parkinson’s Progression Markers Initiative, demonstrating that disease progression follows sex-specific patterns, with rigidity, autonomic dysfunction, and *SNCA* variants being more influential in males, whereas verbal fluency and urinary dysfunction were stronger predictors in females [15]. Likewise, D’Amore et al. [16] employed XGBoost models using Alzheimer’s Disease Neuroimaging Initiative (ADNI) data and found that women exhibited higher predictive weighting of episodic memory decline, while men showed stronger dependence on global cognition and executive function. Together, these studies highlight sex as an easily measurable yet biologically meaningful determinant of neurodegenerative trajectories [16]. When combined with cutaneous inflammatory profiles, which reflect systemic immune activation, such parameters could enhance predictive frameworks for dementia onset and progression, fostering early, personalized, and clinically accessible strategies in neurodegenerative disease surveillance.

While sex and inflammatory pathways provide a plausible biological connection between the skin and brain, the contribution of shared genetic mediators remains insufficiently defined. Recent investigations have revealed overlapping genetic signatures in psoriasis, AD and PD, and emerging links involving rosacea, atopic dermatitis, bullous pemphigoid and dementia. These findings suggest that convergent molecular mechanisms, particularly those governing immune regulation and chronic inflammation, may predispose individuals to both cutaneous and neurological pathology. Thus, genetic susceptibility represents a stable determinant of disease risk, potentially explaining why specific subsets of individuals develop both chronic inflammatory skin disorders and dementia.

In this review, we synthesize current evidence on the genetic overlap between dermatological and neurodegenerative conditions. By highlighting key molecular mediators of the skin–brain axis, we aim to provide mechanistic insights into shared pathogenic circuits and contribute to future perspectives on risk stratification and precision medicine approaches. Furthermore, through this work, we seek to establish a stronger clinical and research connection between inflammatory skin diseases and dementia, advocating that patients with chronic inflammatory dermatoses should be routinely evaluated for cognitive performance. Such early neurocognitive screening could enable timely detection of dementia-related changes and ultimately improve patients’ long-term quality of life.

## 2. Shared Genetic Mediators Linking Inflammatory Skin Diseases and Neurodegeneration

This section synthesizes genomic and transcriptomic data from human studies investigating shared susceptibility loci, pleiotropic variants, and immune regulatory pathways connecting chronic inflammatory skin diseases with Alzheimer’s and Parkinson’s disease. Literature was identified through targeted searches of PubMed, IEEE Xplore, Google Scholar, and ResearchGate, prioritizing human-based genetic and transcriptomic evidence.

### 2.1. Psoriasis and Alzheimer’s Disease

#### 2.1.1. Epidemiology and Comorbidity

Psoriasis is a chronic, immune-mediated inflammatory disorder of the skin with a marked proliferative component, affecting approximately 2–4% of the world’s population. Clinically, it manifests as well-demarcated erythematous plaques covered with silvery-white scales, most commonly involving the extensor surfaces, scalp, and lumbosacral region. The disease arises from a complex interplay between inherited genetic susceptibility and environmental triggers, which sustain immune activation and drive excessive keratinocyte turnover. By far the most prevalent form is plaque psoriasis, accounting for up to 90% of cases, whereas pustular and guttate variants occur less frequently [17,18].

At the immunological level, psoriasis is driven by aberrant activation of innate and adaptive immune pathways, involving dendritic cells, macrophages, and natural killer (NK) cells that initiate local inflammation. Subsequent activation of Th1 and Th17 lymphocytes amplifies the inflammatory cascade through interleukin-17 (IL-17) and tumor necrosis factor-α (TNF-α) signaling. Consequently, targeted biologic agents directed against these cytokines have revolutionized treatment, outperforming conventional systemic therapies and establishing the current standard of care for moderate-to-severe disease [17,18].

Moreover, psoriasis is now recognized as a systemic inflammatory condition, with approximately one-third of patients developing psoriatic arthritis, characterized by joint inflammation, pain, and stiffness. In addition, cardiometabolic and psychiatric comorbidities, including cardiovascular disease, metabolic syndrome, and depression, are frequently observed and are attributed to persistent systemic inflammation [17,18].

Clinical guidelines increasingly emphasize that effective management of psoriasis-associated comorbidities can markedly improve patients’ quality of life. The consequences of chronic systemic inflammation extend beyond traditional associations, emphasizing the importance of identifying additional related conditions and elucidating the biological mechanisms that connect them [19].

Despite major advances in understanding its immunopathogenesis, the precise etiology of psoriasis remains elusive. Current evidence supports a multifactorial origin involving both genetic predisposition and environmental triggers. Major environmental factors include excessive ultraviolet (UV) exposure, medications such as lithium, β-blockers, and imiquimod, smoking, alcohol consumption, dietary factors and obesity, infections (particularly streptococcal), microbial dysbiosis of the skin and gut, and psychological stress. These triggers promote disease onset and progression through pathways involving T-cell activation, cytokine dysregulation (e.g., IL-17/IL-23 axis), and epigenetic modifications that sustain keratinocyte hyperproliferation and chronic inflammation [20].

In addition, several genetic studies have identified multiple susceptibility loci for psoriasis, including *psoriasis susceptibility region 1* (*PSORS1*), *IL12B*, and the *vitamin D receptor* (*VDR*) gene [21,22,23]. Further reports have also implicated polymorphisms in the *apolipoprotein E* (*ApoE*) gene, a locus also strongly associated with AD, supplementary featuring the complex genetic architecture of psoriasis [24]. The *APOE* locus, located on chromosome 19, encodes apolipoprotein E, a protein essential for lipid transport, neuronal repair, and vascular integrity. Three common alleles—ε2, ε3, and ε4—produce isoforms with distinct biological effects. The ε4 allele confers a dose-dependent risk for late-onset AD (LOAD) and atherosclerosis, ε2 appears protective against AD but predisposes carriers to type III hyperlipoproteinemia, whereas ε3 has been proposed to exert a protective influence against psoriasis, as discussed later in this review [25,26,27].

AD is the leading cause of dementia, accounting for 60–80% of cases in the elderly population. More than seven million individuals aged ≥65 years are currently affected in the United States, and this number is projected to nearly double within the next few decades [28]. Neuropathologically, AD is defined by the accumulation of extracellular amyloid-β (Aβ) plaques and intracellular neurofibrillary tangles composed of hyperphosphorylated tau, accompanied by progressive neuronal loss, chronic neuroinflammation, and vascular dysfunction culminating in cognitive decline. Although rare early-onset familial forms result from mutations in *APP*, *PSEN1*, or *PSEN2*, the vast majority of cases are LOAD, in which polymorphisms in *APOE* remain the most robust and reproducible genetic risk factor [29].

The epidemiological association between psoriasis and AD has been increasingly substantiated. In a nationwide Korean cohort including over 535,000 psoriasis patients and 2.6 million age and sex-matched controls, AD occurred in 2.11% of psoriasis patients compared with 1.87% of controls, corresponding to an approximately 13–14% higher relative prevalence and an adjusted hazard ratio (HR) of 1.09 (95% CI 1.07–1.12). The association was strongest in middle-aged adults and absent among patients receiving systemic anti-inflammatory therapy, suggesting that chronic systemic inflammation may contribute to AD pathogenesis [30].

In a recent retrospective cohort study from Germany including nearly 21,000 participants, psoriasis was associated with an increased risk of developing dementia. Over a 15-year follow-up period, 22.0% of patients with psoriasis developed dementia compared with 19.1% of matched controls without psoriasis. This corresponded to an HR of 1.24 and an incidence rate of 15.0 per 1000 person–years in psoriasis patients versus 11.9 per 1000 person–years in controls. In the subgroup with psoriatic arthritis, 18.6% developed dementia compared with 14.3% of non-PsA controls (HR = 1.35). Overall, these findings indicate that individuals with psoriasis have approximately a 24% higher relative risk and a 3% absolute increase in dementia incidence compared with the general population, supporting a modest but statistically significant link between chronic cutaneous inflammation and neurodegenerative comorbidity [14]. These findings support the hypothesis that effective control of inflammatory skin disease could help mitigate neurodegenerative risk.

Therapeutic exposure may further influence this relationship. In the same Korean cohort, patients receiving systemic treatments, including acitretin, methotrexate, cyclosporine, and biologic agents, showed a lower HR for AD compared with untreated individuals (incidence 3.7 vs. 6.5 per 1000 person–years; *p* < 0.0001; HR 0.988 vs. 1.098) [30]. These findings suggest that effective systemic immunomodulation may mitigate neuroinflammatory pathways contributing to AD pathogenesis in psoriasis.

However, findings from a smaller Dutch Rotterdam Study demonstrated no increase, and even a potential reduction, in dementia risk among psoriasis patients. In that cohort, 4.8% of psoriasis patients developed dementia (including AD). However, after multivariable adjustment, the HR was 0.50, indicating roughly half the risk observed in the general population [31]. Taken together, while a large-scale Asian study and a European cohort study report approximately a 15–20% higher risk of AD among individuals with psoriasis, this association is not universally consistent. Such variability likely reflects variations in genetic background, environmental exposures, comorbid burden, and therapeutic practices that collectively modulate systemic inflammation and neurodegenerative susceptibility.

#### 2.1.2. Cytokine Network and Therapeutic Biologics Overlap (IL-17/IL-23/TNF)

Beyond the genetic overlap discussed in this review, emerging immunological evidence indicates that psoriasis and AD are both chronic immune-mediated conditions whose pathophysiology converges along the skin–brain axis through sustained systemic inflammation and overlapping cytokine networks. A key component of this link is the IL-17/IL-23 axis, a central driver of peripheral and central immune activation. In psoriasis, persistent stimulation of Th17 cells results in excessive production of IL-17A and IL-23, which promote keratinocyte proliferation, angiogenesis, and leukocyte recruitment to the skin. In AD, the same cytokine cascade contributes to microglial activation, amyloid-β (Aβ) accumulation, and neuronal apoptosis, thereby perpetuating neuroinflammation and cognitive decline. Experimental data demonstrate that inhibition of the IL-12/23p40 subunit or blockade of IL-17A attenuates microglial activation, reduces Aβ deposition, and improves cognitive performance in AD models [32].

Similarly, TNF-α acts as a shared pro-inflammatory mediator implicated in both psoriasis and AD. Elevated TNF-α contributes to chronic inflammation, immune activation, and neuronal dysfunction through amplification of Aβ and tau pathology, as demonstrated in experimental and clinical studies. According to Decourt et al. [33], TNF-α signaling exacerbates both Aβ and tau pathologies in vivo, while pharmacological inhibition of TNF-α reduces neuroinflammation, decreases Aβ deposition, and improves cognitive performance in animal models and early clinical trials. In parallel, Zhou et al. [34] analyzed more than 56 million electronic health records and reported that treatment with TNF-α inhibitors, including etanercept, adalimumab, and infliximab, was associated with a significantly lower risk of AD among patients with rheumatoid arthritis and psoriasis. Specifically, in psoriasis, etanercept and adalimumab were linked to adjusted odds ratios of 0.47 (95% CI 0.30–0.73) and 0.41 (95% CI 0.20–0.76), respectively, compared with untreated individuals.

Given the growing recognition of inflammation as a shared pathogenic mechanism between psoriasis and neurodegenerative disorders, Levitt et al. [35] in a 2025 Israeli population-based cohort study, provided epidemiological evidence that biologic therapy may reduce the risk of dementia among individuals with psoriasis. Using a national health insurance database, the authors compared elderly psoriasis patients (≥65 years) treated with biologic agents, including IL-17 inhibitors (secukinumab, ixekizumab), IL-23 inhibitors (guselkumab, risankizumab), the IL-12/23p40 inhibitor (ustekinumab), and TNF-α inhibitors (etanercept, adalimumab, infliximab, certolizumab pegol), against matched controls receiving non-biologic systemic therapies such as methotrexate, acitretin, apremilast, or ciclosporin. Over a 10-year follow-up, biologic-treated patients showed a significantly lower incidence of dementia, with an HR of 0.47 (95% CI 0.323–0.699) and an adjusted HR of 0.52, corresponding to roughly a 50% reduction in relative risk compared with conventional therapy. These findings suggest that inhibition of these key inflammatory cytokines may mitigate systemic and neuroinflammatory processes implicated in cognitive decline. However, the study did not specifically assess early initiation of biologics or include cognitive outcomes as primary endpoints, and no randomized clinical trials have yet examined IL-17 or IL-23 inhibitors for dementia prevention or treatment [35].

In summary, converging mechanistic and clinical evidence points to dysregulated cytokine signaling involving IL-17, IL-23, and TNF-α as a shared inflammatory axis linking psoriasis and AD. They further highlight that cytokine-targeted biologic therapies, by suppressing systemic inflammation, could offer dual benefits, controlling cutaneous disease activity and potentially reducing neurodegenerative risk, thereby warranting further mechanistic and interventional studies to clarify their long-term neuroprotective potential.

#### 2.1.3. Candidate Gene Evidence (APOE and IL12B)

There is evidence suggesting that *APOE* polymorphisms also contribute to dermatological disease, since carriers of ε2 and ε4 alleles have been reported to show increased risk of psoriasis [27]. In 1997, in one of the earliest investigations, Furumoto et al. [24] demonstrated that the ε2 allele was significantly overrepresented in Japanese patients with psoriasis vulgaris compared with healthy controls, via analytical isoelectric focusing followed by immunoblotting [24]. This finding was not left unnoticed and further established later by a candidate-gene meta-analysis by Han et al. [27], who pooled data from seven case–control studies including ~1000 psoriasis patients and ~1000 controls from Asian and European populations. This analysis revealed that ε2 confers increased susceptibility to psoriasis, whereas ε3 and the ε3/ε3 genotype are protective. Although ε4 was not significantly associated with psoriasis overall, subgroup analyses indicated that in European cohorts it correlated with increased risk and disease severity, findings not replicated in Asian populations. These ethnic discrepancies likely reflect geographic variation in allele frequencies: ε3 is globally predominant, particularly in Mediterranean populations, while ε4 exhibits a north–south decline across Europe and a similar gradient across China. These patterns parallel established differences in ε4-associated AD risk, underscoring the context-dependent effects of *APOE* [27]. Together, these findings suggest that *APOE* polymorphisms exemplify how lipid metabolism, vascular health, and inflammatory pathways intersect to influence both cutaneous and neurodegenerative disease.

Mechanistic explanations further strengthen this connection. ApoE2, with the lowest receptor-binding affinity, reduces lipoprotein clearance and predisposes to hyperlipidemia, a frequent comorbidity in psoriasis [36]. ApoE4, although a major risk factor for AD through its atherogenic and pro-inflammatory effects, has paradoxically been linked to higher circulating vitamin D levels. Given the central role of vitamin D in keratinocyte proliferation, epidermal differentiation, and neuroimmune modulation, this paradox may partly account for the variable association of ε4 with psoriasis. Together, these pleiotropic and allele-specific effects position *APOE* as a central mediator at the interface of cutaneous inflammation and neurodegeneration. Limitations of current analyses include the relatively small number of studies (*n* = 7), modest sample sizes, restriction to Asian and European cohorts, and potential publication bias. Larger, multi-ethnic investigations are needed to refine these associations and elucidate the molecular mechanisms through which ApoE influences both psoriasis and AD [27].

Additionally, the *IL12B* gene, which encodes the p40 subunit common to IL-12 and IL-23, has emerged as an important genetic factor in both psoriasis and AD, albeit through different biological pathways [37,38,39,40]. In psoriasis, the role of *IL12B* was demonstrated in a large multi-stage genetic association study performed in white North American cohorts. After screening more than 25,000 SNPs and replicating the findings in independent populations, *IL12B* was confirmed as a susceptibility locus. Two polymorphisms, rs3212227 in the 3′ UTR and rs6887695 upstream of the coding region, were consistently associated with disease, forming haplotypes that either increased risk (OR ~1.40) or conferred protection (OR ~0.58). Diplotype analysis showed a clear gene-dose effect, as individuals homozygous for the risk haplotype were at substantially higher risk, while homozygosity for the protective haplotype reduced susceptibility. The scale of the study, replication across several cohorts, and alignment of the genetic findings with the therapeutic success of p40-targeting biologics lend strong support to the association. At the same time, the absence of functional assays to clarify the mechanisms of these variants and the focus on European ancestry populations leave some questions regarding their biological effect and generalizability [38].

The therapeutic importance of *IL12B* in psoriasis was highlighted by early clinical trials that targeted its encoded p40 subunit, common to IL-12 and IL-23. In 2008, the pivotal clinical trial PHOENIX that enrolled 766 patients (in PHOENIX 1) with moderate-to-severe plaque psoriasis studied the efficacy and safety of ustekinumab, a monoclonal antibody against the p40 protein. It resulted in rapid and substantial clinical improvements and by week 12, approximately two-thirds of patients receiving ustekinumab achieved at least 75% improvement in the Psoriasis Area and Severity Index (PASI 75), compared with only 3% of patients on placebo. Withdrawal from treatment led to gradual disease recurrence, while re-initiation restored therapeutic benefit within weeks [39]. Ustekinumab is now widely used for the management of several chronic inflammatory disorders, including psoriasis, psoriatic arthritis, and inflammatory bowel disease. Recent epidemiological data, as seen above, further suggest that its use is associated with a significantly lower incidence of dementia, supporting a potential neuroprotective effect linked to systemic cytokine modulation [35,41].

Complementary evidence for the *IL12* comes from a large case–control study in a Northern Han Chinese population, which investigated *IL12A* and *IL12B* variants in 1133 patients with late-onset AD and 1158 controls. The *IL12B* polymorphism rs3212227 was associated with disease risk, with carriers of the GG or GT genotypes showing a lower susceptibility than TT homozygotes. Combined analyses indicated that *IL12A* and *IL12B* variants exerted additive effects, consistent with the coordinated requirement of p35 and p40 subunits for IL-12 activity. The study’s size, careful adjustment for confounding factors such as *APOE ε4* status, and the novelty of linking *IL12B* to AD provide credibility, though the single-ethnicity design raises concerns about population specificity and functional validation remains lacking. Moreover, the association was not detected in prior European-based Genome-Wide Association Studies (GWAS), reflecting possible differences in allele frequencies between populations [40].

Taken together, these human studies suggest that *IL12B* serves as a shared genetic mediator connecting peripheral autoimmune disease and central neurodegeneration. In psoriasis, *IL12B* promotes pathogenic IL-23 signaling that drives Th17-mediated skin inflammation, whereas in AD, *IL12B* variation influences IL-12–dependent neuroinflammatory pathways and modulates susceptibility to cognitive decline. A recent mechanistic study in mouse models further supports this conclusion by showing that IL12B-derived p40 drives IL-12 signaling in both neurons and oligodendrocytes, exacerbating amyloid pathology and neurodegeneration [42].

Together, these convergent findings highlight *IL12B* and *APOE* as a context-dependent but biologically significant link between psoriasis and AD, emphasising the broader importance of inflammatory cytokine pathways as potential therapeutic targets across immune-mediated and neurodegenerative conditions.

#### 2.1.4. Immune Loci from GWAS (HLA-DRB5 and Related Variants)

Beyond candidate-gene associations, genome-wide association approaches have reinforced the notion of a shared genetic architecture. A pleiotropy-informed GWAS framework analyzing summary statistics from more than 100,000 individuals tested whether variants implicated in immune-mediated diseases also predispose to AD. Among the autoimmune conditions assessed, psoriasis showed a significant overlap with AD through a variant in the human leukocyte antigen (HLA) region. Specifically, SNP rs2516049 on chromosome 6, near *HLA-DRB5*, was associated with both psoriasis and AD, with a consistent direction of effect. This variant also correlated with greater neurofibrillary tangle burden and accelerated cognitive decline, linking it to AD pathology. Gene expression analyses revealed that while *HLA-DRB5* itself was not differentially expressed in AD brains, its paralog *HLA-DRA* was significantly upregulated, further implicating HLA-mediated immune dysregulation. The strength of this study lies in its use of large GWAS datasets, rigorous pleiotropy-informed statistical methods, and integration with neuropathological and transcriptomic validation. Yet, the inherent complexity of the HLA locus complicates pinpointing causal variants, reliance on clinically defined AD diagnoses introduces heterogeneity, and analyses focused primarily on European cohorts, limiting generalizability. Nevertheless, the identification of rs2516049 near *HLA-DRB5* provides strong evidence that psoriasis and AD share immune-related genetic susceptibility [43]. This highlights the pivotal role of HLA-mediated antigen presentation in bridging autoimmune and neurodegenerative processes, although the complexity of this locus still obscures precise causal mechanisms.

#### 2.1.5. Transcriptomic Regulators (ZNF384 and Network-Level Dysregulation)

More recent bioinformatic investigations have advanced these findings by exploring shared transcriptional networks. Using publicly available datasets, researchers compared gene expression profiles in psoriatic lesioned skin and hippocampal tissue from AD patients. Differential expression analysis identified over one hundred Differentially Expressed Genes (DEGs) common to both conditions, many of which mapped to inflammatory and metabolic pathways. Pathway enrichment confirmed the involvement of IL-17 and TNF signaling, PPAR signaling, arachidonic acid metabolism, and nitrogen metabolism, pathways well-established in psoriasis and increasingly recognized in AD pathogenesis. Among the shared DEGs, five transcription factors (*PPARG*, *ZFPM2*, *ZNF415*, *HLX*, and *ANHX*) emerged as potential regulators. Promoter sequence analysis using JASPAR and UCSC Genome Browser revealed *ZNF384* as a likely master regulator, with predicted binding sites in the promoters of *PPARG*, *ZNF415*, *HLX*, and *ANHX*. Through its influence on JAK–STAT signaling, *ZNF384* may perpetuate a feedback loop of inflammation and metabolic imbalance, thereby driving pathology in both skin and brain. Clinically, the observation that biologics targeting IL-17 or TNF not only improve psoriasis but may also lower AD risk provides further support for the relevance of these shared mechanisms. The main strength of this study lies in its systems biology approach, which integrates transcriptomic data across diseases to identify convergent regulators and pathways. However, its reliance on in silico predictions, relatively modest dataset sizes, and the lack of experimental validation highlight the need for future functional studies to confirm the role of *ZNF384* and related regulators [44]. These transcriptional regulators emphasize that beyond single-gene associations, network-level dysregulation of inflammatory and metabolic signaling may represent an unifying theme in psoriasis and AD.

#### 2.1.6. Shared Genetic Mediators Linking Psoriasis and Alzheimer’s Disease

Collectively, as seen in Table 1 below, converging lines of evidence identify *APOE*, *HLA*, *IL12B*, and transcriptional regulators such as *ZNF384* as genetic links bridging psoriasis and AD. These findings feature the relevance of shared inflammatory and metabolic pathways in cutaneous and neurodegenerative disease. However, limitations in study design, including small sample sizes, ethnic biases, and reliance on in silico methods, highlight the need for larger, multi-ethnic, and functionally validated investigations. Deciphering the molecular mechanisms underlying these shared mediators delineates a biologically plausible axis whereby allele- and pathway-specific perturbations in lipid metabolism, cytokine signaling, and antigen presentation co-promote psoriatic inflammation and AD-related neurodegeneration. Thereby, defining testable targets (e.g., p40/IL-23, IL-17, TNF) for cross-disease therapeutic exploration could yield novel biomarkers and therapeutic targets with cross-disease relevance, advancing precision medicine at the interface of dermatology and neurology.

#### 2.1.7. Possible Disease-Specific Mechanistic Axis Linking Psoriasis and Alzheimer’s Disease

In summary, convergent human genetic and transcriptomic data delineate disease-specific mechanistic links between psoriasis and AD centered on lipid–immune crosstalk and antigen-presentation pathways. Candidate-gene data indicate that *APOE* polymorphisms modulate risk in both conditions: ε2 and ε4 have been reported to increase psoriasis susceptibility (with ε3 protective), mirroring ε4-driven AD risk and suggesting allele-specific effects on lipid handling, vascular integrity, and inflammation. Mechanistically, reduced receptor binding by ApoE2 can promote hyperlipidemia common in psoriasis, while ApoE4’s pro-inflammatory profile, and its paradoxical association with higher vitamin-D levels relevant to keratinocyte biology, highlights context-dependent pleiotropy [24,27]. Complementing this, psoriasis susceptibility variants in *IL12B* (rs3212227, rs6887695) strengthen *IL-23/Th17* signaling that sustains cutaneous inflammation and align with the clinical efficacy of p40 blockade (ustekinumab), whereas in AD, *IL12B* variation tracks with *IL-12*–dependent neuroinflammatory cascades and experimental evidence that p40 can exacerbate amyloid pathology in neural cells [38,39,40]. Beyond candidates, pleiotropy-informed GWAS identify shared HLA architecture (rs2516049 near *HLA-DRB5*) with concordant effects on psoriasis and AD and correlations with tau pathology and cognitive decline, implicating antigen presentation in both skin and brain [43]. Network-level analyses further converge on inflammatory–metabolic programs (IL-17/TNF, PPAR, arachidonic and nitrogen metabolism) and nominate transcriptional regulators, most notably ZNF384 via JAK–STAT coupling, as putative cross-disease drivers [44].

### 2.2. Psoriasis and Parkinson’s Disease

#### 2.2.1. Epidemiology and Molecular Evidence

PD is the second most prevalent neurodegenerative disorder, affecting more than 1% of individuals globally over the age of 65, with prevalence projected to nearly double by 2030 [45]. The hallmark pathology involves progressive loss of dopaminergic neurons in the Substantia nigra pars compacta, leading to striatal dopamine depletion and consequent disruption of basal-ganglia motor circuits. Besides, abnormal intraneuronal aggregation of α-synuclein in Lewy bodies is implicated in synaptic dysfunction, axonal degeneration and the multisystem neurodegeneration [46].

In addition to its characteristic motor features, PD is frequently accompanied by non-motor manifestations, among which cognitive impairment is one of the most common and may occur at any stage of the disease. The mechanisms underlying cognitive decline in PD remain incompletely understood. While cortical involvement by Lewy body pathology and Alzheimer-type changes appear central, additional interacting molecular and inflammatory pathways are increasingly recognized. Cognitive impairment may emerge before the onset of motor symptoms, coincide with initial diagnosis, or manifest years to decades thereafter [47,48]. Its trajectory is highly variable in terms of severity, cognitive domains affected, and rate of progression.

Longitudinal cohort studies have consistently demonstrated that patients with PD carry a substantially increased risk, approximately 2.5- to 6-fold higher, of developing dementia compared with age-matched individuals without PD. A key challenge remains the absence of disease-modifying therapies to prevent or delay cognitive decline in PD, highlighting the urgent need for reliable biomarkers to predict cognitive decline and identify patients at greatest risk for early or rapid deterioration [49]. Early identification of such patients is essential to enable timely intervention and improve long-term outcomes.

Accumulating evidence supports a biological and molecular continuum between the skin and the brain in PD. Key PD-related genes, including *PINK1*, *LRRK2*, and *PARKIN*, which regulate mitochondrial integrity, autophagy, and oxidative homeostasis in dopaminergic neurons, exhibit comparable dysfunction in patient-derived skin fibroblasts. These peripheral cells display inflammatory, metabolic, and lysosomal disturbances analogous to those observed in neuronal tissue, indicating that systemic mitochondrial and immune stress propagates beyond the central nervous system. Furthermore, the presence of phosphorylated α-synuclein aggregates in cutaneous nerve fibers provides histopathological evidence that neurodegenerative protein misfolding extends into the periphery, reinforcing the notion that the skin mirrors central neuropathology [50].

Complementary insights arise from induced pluripotent stem cell (iPSC) models generated from PD fibroblasts. When reprogrammed and differentiated into dopaminergic neurons, these cells reproduce hallmark Parkinsonian phenotypes, including reduced neurite arborization, impaired autophagic flux, and accumulation of autophagic vacuoles, faithfully reflecting the pathology of the substantia nigra [51]. This convergence of molecular, histological, and functional evidence substantiates a functional and bidirectional inflammatory skin–brain axis, wherein cutaneous molecular alterations not only mirror but may also contribute to the broader neurodegenerative processes underlying PD [50].

Within this framework, increasing attention has focused on shared pathogenic mechanisms between PD and inflammatory skin diseases such as psoriasis. The pathogenic link between psoriasis and PD is supported by convergent evidence implicating chronic neuroinflammation and systemic immune activation in both conditions. Both are characterized by persistent elevations of proinflammatory cytokines, including TNF-α, IL-1β, IL-6, IL-12, and interferon-γ, which are upregulated not only in psoriatic skin but also in the brains and cerebrospinal fluid of patients with PD. Dysregulation of anti-inflammatory cytokines, particularly IL-10, may further perpetuate the inflammatory milieu common to both diseases. Activation of microglial cells in PD parallels immune activation in psoriatic lesions, leading to oxidative stress, liberation of reactive oxygen and nitrogen species, and neuronal injury. Both conditions also involve adaptive immune responses mediated by TH1 and TH17 cells, driven by IL-12 and IL-23, respectively, while increased circulating TH17 and myeloid-derived suppressor cells have been observed in PD, reflecting an immune signature analogous to that of psoriasis [52].

A systematic review and meta-analysis by Ungprasert et al. [53] comprehensively evaluated this association across large population-based datasets from Europe and Asia. The pooled analysis demonstrated that patients with psoriasis have a 38% higher relative risk of developing PD compared with controls [53]. Further epidemiological evidence from a large nationwide Korean cohort confirmed this association, reporting that individuals with psoriasis developed PD more often than matched controls, roughly 0.77 versus 0.67 cases per 1000 person–years. This translated to an adjusted hazard ratio of 1.09 (95% CI 1.03–1.12), meaning that after accounting for factors such as age, sex, and comorbidities, patients with psoriasis had an approximately 9% higher risk of developing PD compared to those without psoriasis. Importantly, the excess risk was mainly observed in patients who did not receive systemic anti-inflammatory therapy, while those treated with agents such as acitretin, methotrexate, or cyclosporine showed a lower incidence of PD. By contrast, TNF-α inhibitors did not significantly affect risk. Of note, acitretin, a retinoid X receptor (RXR) agonist, may exert neuroprotective effects through RXR-mediated support of dopaminergic neuron survival and differentiation, suggesting that systemic immune modulation could influence neurodegenerative trajectories [52].

These findings collectively indicate that systemic immune regulation can impact neurodegenerative outcomes, linking dermatological and neurological pathologies through shared inflammatory mechanisms, although the precise genetic underpinnings remain to be elucidated. Based on a recent Mendelian randomization study, psoriasis appears to exert a causal influence on the progression of PD through shared genetic and inflammatory mechanisms. Using GWAS summary statistics from over 33,000 individuals with psoriasis and large GWAS datasets on PD onset and progression, the investigators employed psoriasis-associated genetic variants as instrumental variables to test whether genetic liability to psoriasis causally affects PD outcomes. The analysis demonstrated that individuals genetically predisposed to psoriasis exhibit a faster rate of PD progression, with increased risks of developing dementia and depression during the disease course. These findings provide genetic-level evidence of a functional connection between psoriasis and PD, supporting the hypothesis that systemic immune dysregulation and chronic inflammation act as convergent biological pathways driving both cutaneous and neurodegenerative processes [54]. In this review, we further explore these shared genetic and molecular mechanisms to clarify how common inflammatory and mitochondrial pathways may link psoriasis and PD within a broader skin–brain axis framework.

#### 2.2.2. Shared Pleiotropic Loci (SETD1A and BC070367)

Beyond immunological overlap, the genetic overlap between chronic inflammatory skin disorders and neurodegeneration has become an area of increasing interest. A pleiotropy-informed GWAS systematically evaluated shared susceptibility loci between PD and seven autoimmune conditions, including psoriasis, across more than 138,000 individuals of European ancestry. Using a conjunction false discovery rate framework, the investigators identified 17 independent pleiotropic loci with dual associations to PD and at least one autoimmune disease [55].

Within psoriasis, two loci emerged with evidence of shared genetic risk. The first was *SETD1A* (rs11640961), which demonstrated opposite directions of effect in psoriasis and PD. The second, *BC070367* (rs1975974), showed concordant effects across both conditions. Although these signals were replicated in independent datasets, the degree of enrichment was weaker than that observed for Crohn’s disease or ulcerative colitis, where overlap with PD was more robust. Still, these findings suggest that psoriasis and PD may converge, at least partially, on common genetic pathways linked to immune regulation [55].

A major strength of this study lies in its genome-wide, cross-phenotype design, which allowed the detection of pleiotropic loci below conventional genome-wide significance thresholds. The use of large, well-characterized datasets and replication in independent cohorts strengthens the validity of the findings. Moreover, functional analyses revealed that many pleiotropic variants mapped to immune-related pathways, lending biological plausibility to an immune-mediated link between psoriasis and PD [55].

Nonetheless, several limitations should be noted. The genetic overlap between psoriasis and PD was modest, with only two loci identified, and the effect directions were inconsistent. The analyses were also limited to individuals of European ancestry, which may restrict applicability to other populations. In addition, the complex linkage disequilibrium structure of immune-related regions complicates the identification of true causal variants. Finally, pleiotropy analyses establish statistical overlap but do not by themselves confirm mechanistic causality [55].

Overall, as seen in Table 2 below, these findings provide preliminary evidence for a genetic connection between psoriasis and PD, centered on loci such as *SETD1A* and *BC070367*.

#### 2.2.3. Possible Disease-Specific Mechanistic Axis Linking Psoriasis and Parkinson’s Disease

Taken together, emerging genomic evidence points to a modest yet biologically meaningful overlap between psoriasis and PD, suggesting potential disease-specific mechanistic links. *SETD1A*, a histone methyltransferase involved in chromatin remodelling and transcriptional regulation, has been implicated in neuronal development and immune gene expression, while BC070367, a non-coding RNA locus, may influence regulatory networks affecting cytokine signaling [55]. The opposing and concordant effects of these variants in psoriasis and PD, respectively, feature the complexity of their functional roles but also highlight a possible interface where immune-mediated mechanisms intersect with neurodegenerative vulnerability. Although the extent of genetic overlap remains limited, these findings reinforce the concept that chronic systemic inflammation and immune signaling constitute a shared biological substrate between psoriasis and PD. Future studies integrating functional genomics, epigenetics, and multiomic analyses will be crucial to unravel how these loci modulate disease pathways and to define novel biomarkers or therapeutic targets relevant to both dermatology and neurology.

## 3. Biological and Genetic Bridges with Other Inflammatory Dermatoses

This section extends the scope of genetic analysis beyond psoriasis to include other inflammatory and autoimmune dermatoses, mainly rosacea, atopic dermatitis, and bullous pemphigoid, that have been associated with an increased risk of neurodegenerative disease. Collectively, these conditions illustrate distinct yet convergent biological pathways through which chronic cutaneous inflammation, vascular dysregulation, and immune activation may influence central nervous system homeostasis. While psoriasis remains the most genetically substantiated model, emerging data from rosacea and atopic dermatitis highlight neurovascular and barrier-mediated mechanisms, whereas bullous pemphigoid exemplifies an autoimmune cross-reactivity model. By integrating findings from genetic, proteomic, and immunologic studies, this section aims to delineate the shared and disease-specific mechanisms underpinning the broader skin–brain axis.

### 3.1. Rosacea and Dementia

#### 3.1.1. Epidemiology and Molecular Evidence

Rosacea is a chronic, relapsing inflammatory disorder of the skin with heterogeneous clinical manifestations, typically involving the central face. Globally, rosacea affects more than 5% of the population, with peak incidence between 30 and 50 years of age, a female predominance, and greater prevalence among individuals of lighter phototypes, where rates may exceed 10% [56,57]. Core features include recurrent flushing, persistent erythema, telangiectasia, and inflammatory papules or pustules, with four recognized clinical subtypes, erythematotelangiectatic, papulopustular, phymatous, and ocular, that frequently overlap and evolve over time. Ocular disease occurs in up to three-quarters of affected individuals and is characterized by irritation, foreign-body sensation, photophobia, and visual disturbance. Beyond its visible manifestations, rosacea imposes a substantial psychosocial burden, contributing to anxiety, depression, and impaired quality of life. Although traditionally regarded as a skin-limited condition, increasing evidence implicates rosacea in a spectrum of systemic comorbidities, including neurological, cardiovascular, gastrointestinal disorders, as well as dementia [56,57].

The skin–brain axis linking rosacea and dementia is supported by convergent inflammatory, proteolytic, and innate immune mechanisms. Rosacea is characterized by upregulation of matrix metalloproteinases (MMPs), notably MMP-1, MMP-3, and MMP-9, and antimicrobial peptides (AMPs) such as cathelicidin (LL-37), which exert potent pro-inflammatory and vasoactive effects within the skin microenvironment. Analogously, in neurodegenerative diseases such as AD, MMP dysregulation and aberrant adenosine monophosphate (AMP) activity contribute to neuronal injury and amyloid pathology. Elevated cerebrospinal fluid concentrations of MMP-3 and MMP-9 have been correlated with both the duration and severity of AD, suggesting their involvement in BBB disruption, amyloid precursor protein processing, and neuroinflammatory amplification. Moreover, Aβ itself functions as an AMP with intrinsic pro-inflammatory and cytotoxic properties, mirroring the innate immune activation observed in rosacea. These shared molecular mediators highlight a plausible biological continuum between cutaneous and cerebral inflammation, providing mechanistic support for epidemiological evidence linking rosacea with increased dementia risk [58].

Epidemiological evidence indicates that the prevalence of rosacea in the general population varies widely, ranging from 1% to 20%, depending on diagnostic criteria, geographic region, and study design [59]. In one of the first clinical investigations exploring the association between rosacea and PD, conducted at the University Hospital Halle in Germany, rosacea was diagnosed in 18.6% of 70 patients with PD, while 31.9% exhibited facial flushing, a neurovascular manifestation that frequently overlaps with rosacea [60]. This substantially higher occurrence relative to general population estimates suggests a potential disease-specific predisposition and supports the hypothesis that the neurogenic and inflammatory dysregulation characteristic of PD may enhance cutaneous susceptibility.

Almost 15 years later, two large nationwide Danish registry studies by Egeberg and colleagues provided robust epidemiological evidence linking rosacea with neurodegenerative diseases. In the first study, including more than 4.6 million adults, individuals with rosacea exhibited almost a twofold higher risk of developing PD compared with those without rosacea, with incidence rates of 0.12 versus 0.06 cases per 1000 person–years [61]. In the companion study, encompassing over 82,000 patients with rosacea among 5.5 million participants, the investigators reported a 25% increased risk of AD and a modest 7% increase in overall dementia, particularly among individuals older than 60 years [58]. Collectively, these findings suggest that the chronic inflammatory environment and immune dysregulation characteristic of rosacea may extend beyond the skin and contribute to neurodegenerative processes through shared pathogenic pathways.

#### 3.1.2. Proteomic and Transcriptomic Overlaps (SNCA, GSK3B, HSPA8)

Recent studies suggest that rosacea shares biological pathways with dementia, although a direct germline genetic link has not been demonstrated [62,63,64]. An integrated proteomics–phosphoproteomics analysis of rosacea skin quantified 3874 proteins and showed that both lesional and clinically non-lesional skin are enriched for inflammatory (e.g., neutrophil activation, IL-12 signaling) and axon-extension programs versus healthy skin. Notably, proteins commonly implicated in neurodegeneration were increased in rosacea, including α-synuclein (SNCA), HSPA8, and GSK3B, while protein-level pathway enrichment flagged AD- and PD-related pathways. Clinically, SNCA positively tracked with CEA (Clinician’s Erythema Assessment), and neutrophil markers ELANE and S100A family proteins correlated with global disease severity (Investigator’s Global Assessment). Phosphoproteomics further implicated activated transcription-factor/kinase networks linked to clinical scores and showed MAPK14 phosphorylation associating with erythema and AKT1 with inflammation. These findings indicate shared protein-level pathway signatures between rosacea and dementia biology but do not demonstrate a shared germline genetic risk [62].

A complementary line of evidence comes from a Greek case–control study that explored whether the *TACR3* promoter polymorphism rs3733631 (C/G), previously linked to neurogenic inflammation, might connect rosacea to neurological disease. While no overall association was found between rosacea and the variant, the G allele and G-carrying genotypes (C/G or G/G) were significantly enriched in papulopustular rosacea, particularly among male patients, whereas erythematotelangiectatic disease showed only borderline enrichment. Because neurokinin B–*TACR3* signaling interacts with dopaminergic and autonomic circuits implicated in PD, the authors proposed that this pathway could represent a biological point of convergence rather than a proven shared genetic risk factor. Importantly, no PD cohort was genotyped, and issues such as small sample size and deviation from Hardy–Weinberg equilibrium in patients warrant cautious interpretation. These preliminary findings suggest that specific genetic variants may influence rosacea subtypes through neurovascular mechanisms, but they do not yet establish a direct genetic bridge to dementia or other neurodegenerative disorders [63].

#### 3.1.3. Regulatory Hubs (PPARG, STAT4, RORA)

Cross-tissue systems biology has further advanced the connection between rosacea and AD. A bioinformatics and network-pharmacology study sought to delineate shared molecular programs between rosacea skin and AD hippocampus and to nominate candidate therapeutics acting on those programs. By integrating human gene-expression data from the GEO (Gene Expression Omnibus) database, the authors identified 747 overlapping differentially expressed genes (DEGs) enriched for immune/inflammatory, lipid-metabolic, and vascular/angiogenic pathways; transcription-factor. Network analysis highlighted hubs such as *PPARG*, *STAT4*, and *RORA*, which then guided drug-gene queries that prioritized melatonin and yielded 19 intersection targets with supportive docking signals [64].

To functionally explore this prediction, the investigators turned to a rosacea-like mouse model, where melatonin treatment reduced lesion area and thickness, immune-cell infiltration (CD4^+^ T cells, F4/80^+^ macrophages), keratinocyte cytokine expression, NF-κB activation, endothelial chemotaxis/migration, and microvessel density. Together, these findings suggest that rosacea and AD share convergent transcriptomic signatures, particularly NF-κB/IL-17/TNF-driven inflammation and vascular remodelling, while pointing to melatonin as a potential modulator. However, the evidence rests on expression overlap, regulatory inference, drug-target enrichment, and validation in an animal model, rather than human genomic data, and therefore does not establish a direct genetic association between rosacea and AD and thus cannot be taken as proof of a direct genetic connection between the two disorders [64]

#### 3.1.4. Possible Disease-Specific Mechanistic Axis Linking Rosacea and Dementia

A potential disease-specific mechanistic axis linking rosacea with dementia through convergent inflammatory, neurovascular, and proteostatic pathways is supported via emerging molecular and clinical evidence. Rosacea exhibits persistent activation of innate immune and proteolytic cascades, particularly MMPs (MMP-1, MMP-3, and MMP-9) and antimicrobial peptides such as cathelicidin (LL-37), that promote chronic neurogenic inflammation, endothelial dysfunction, and oxidative stress. These same mediators are implicated in the pathophysiology of AD, where MMPs overactivity contributes to Aβ aggregation, BBB disruption, and progressive neuroinflammation [58]. Beyond these canonical inflammatory links, recent proteomic and transcriptomic studies reveal deeper biological parallels. Rosacea lesional and non-lesional skin display upregulation of neurodegeneration-related proteins, including SNCA, HSPA8, GSK3B, key regulators of protein misfolding, autophagy, and tau phosphorylation in AD and PD. These alterations, together with enrichment of MAPK14 and AKT1 phosphorylation networks, delineate a cutaneous signature of dysregulated stress-response and axonal-extension pathways that recapitulate central nervous system neurodegenerative programs [62].

Integrative bioinformatics and network pharmacology analyses combining transcriptomic datasets from AD and rosacea identified several shared regulatory hubs, including *PPARG*, *STAT4*, and *RORA*, which converge on NF-κB, IL-17, and TNF signaling pathways implicated in chronic inflammation and vascular remodelling. Further, experimental validation in rosacea-like murine models demonstrated that melatonin treatment significantly reduced inflammatory cell infiltration, downregulated keratinocyte cytokines (such as IL-6, IL-8, and IL-1β), and suppressed microvascular proliferation, supporting the pathogenic relevance of these shared pathways [64]. Although these findings suggest the presence of a common inflammation–vascular axis linking peripheral and central neuroinflammatory processes, several limitations must be acknowledged. The analyses were entirely in silico and cross-sectional, based on publicly available transcriptomic datasets derived from heterogeneous tissue sources and small sample sizes, and the experimental validation relied on murine models that may not fully recapitulate human neurovascular complexity. Moreover, the absence of longitudinal, proteomic, or germline genetic validation precludes causal inference and limits the ability to determine whether the observed transcriptomic convergence represents a true shared etiology or parallel downstream inflammatory responses [64].

Collectively, these data point toward a common inflammation–axon–vascular module bridging peripheral and central tissues, wherein chronic cutaneous inflammation and neurovascular dysregulation in rosacea may mirror or even propagate mechanisms of neurodegeneration. While current evidence substantiates shared molecular and transcriptomic pathways, a definitive germline genetic overlap has not yet been demonstrated, accentuating the need for cross-disciplinary genomic and mechanistic studies to clarify whether rosacea constitutes an early systemic manifestation or comorbid amplifier of dementia pathobiology.

### 3.2. Atopic Dermatitis and Dementia

#### 3.2.1. Epidemiological Evidence

Atopic dermatitis is the most prevalent chronic inflammatory skin disorder and a prototypical form of eczema. It is characterized by intense pruritus, xerosis, eczematous lesions, and lichenification, typically beginning in early childhood but often persisting or recurring throughout life. Epidemiological data indicate that atopic dermatitis affects approximately 10–30% of children and 2–10% of adults in industrialized nations, with prevalence having increased two- to three-fold in recent decades. The disease results from a complex interaction between genetic predisposition and environmental triggers that disrupt epidermal barrier integrity and immune homeostasis. Loss-of-function mutations in *FLG*, encoding filaggrin, an essential barrier protein responsible for epidermal hydration and mechanical stability, are among the most strongly implicated genetic risk factors, present in up to 30% of patients. Such variants not only predispose to atopic dermatitis but also confer susceptibility to ichthyosis vulgaris, allergic rhinitis, and keratosis pilaris. Clinically, atopic dermatitis is part of the “atopic march,” the sequential or concurrent manifestation of atopic disorders including asthma and allergic rhinoconjunctivitis [65,66].

Earlier epidemiological investigations suggested that individuals with atopic eczema may face nearly a two-fold higher risk of developing dementia; however, these studies were limited by small samples and self-reported diagnoses [67,68]. In 2022, a large UK population-based cohort of more than 1.7 million adults aged 60–99 years found that those with eczema had a 27% higher risk of dementia than those without the condition. This association, observed for both Alzheimer’s and vascular dementia, increased with eczema severity and persisted after adjustment for comorbidities and corticosteroid use, with dementia typically diagnosed about five years after eczema onset [7].

Despite these consistent epidemiological findings, a 2025 Mendelian randomization analysis including more than 860,000 individuals found no statistically significant causal genetic relationship between atopic dermatitis and dementia [69]. This suggests that the observed epidemiological link may reflect secondary immunoinflammatory or vascular mechanisms rather than direct shared genetic susceptibility. However, the absence of a causal signal at the population level does not exclude the presence of gene or pathway-specific interactions that remain undetected in genome-wide analyses. Mendelian randomization relies primarily on common SNPs and may overlook the influence of rare or regulatory variants acting through intermediate biological networks.

#### 3.2.2. Biological Connection: Immune-Inflammatory and Vascular Pathways

The biological connection between atopic dermatitis and dementia is increasingly understood to involve a complex interplay between systemic type 2-driven inflammation, neuroimmune activation, and vascular dysfunction. In atopic dermatitis, chronic elevation of cytokines such as IL-4, IL-13, and IL-6 perpetuates a Th2-skewed immune response that extends beyond the skin, leading to persistent systemic inflammation. Circulating cytokines and immune mediators can compromise the structural and functional integrity of the BBB, promoting leukocyte infiltration, microglial activation, and sustained neuroinflammatory signaling within the central nervous system. In particular, IL-6 has been shown to upregulate the expression of Aβ precursor protein, while persistent systemic inflammation impairs the microglial clearance of Aβ peptides, resulting in their pathological accumulation. This Aβ burden, together with chronic glial activation, enhances oxidative stress and synaptic dysfunction, linking peripheral inflammation to amyloidogenic neurodegeneration. Concomitantly, inflammatory endothelial activation and vascular remodeling, driven by cytokine and mast-cell-mediated pathways, induce endothelial dysfunction characterized by increased adhesion molecule expression, vascular permeability, and oxidative stress. These alterations impair cerebral perfusion and oxygen delivery, contributing to neuronal energy imbalance and progressive cognitive decline. Together, these findings suggest that the skin–brain axis in atopic dermatitis operates through an immunoinflammatory–vascular continuum, wherein chronic peripheral inflammation propagates cerebrovascular injury, Aβ accumulation, and neurodegenerative mechanisms underlying dementia [69].

#### 3.2.3. FLG Rare Variants and Barrier Dysfunction

Adding to these epidemiological and biological observations, recent genetic evidence suggests that atopic dermatitis and AD may share susceptibility factors. Xiong et al. [70] aimed to explore whether rare genetic variants contribute to AD risk beyond the well-established common loci. Using data from the ADNI, the investigators applied a rare variant association framework to systematically scan for genes carrying rare exonic variants enriched in AD cases compared with controls. Among the top signals, they identified *FLG*, the gene encoding filaggrin, a key epidermal barrier protein and a well-known risk factor for atopic dermatitis, as a candidate gene in AD. The identification of *FLG* in this context was unexpected, as it is classically associated with skin barrier dysfunction, yet its emergence in dementia genetics suggests that genes central to cutaneous biology may also contribute to neurodegenerative processes [70].

#### 3.2.4. Filaggrin–BACE1 Interaction and Amyloid Biology

To investigate how *FLG* variants might contribute to AD, the study incorporated computational modeling. Docking and molecular dynamics simulations showed that filaggrin, the protein encoded by *FLG*, can bind to the β-site APP-cleaving enzyme 1 (the protein encoded by the *BACE1* gene), a key enzyme responsible for generating Aβ. Importantly, a rare *FLG* variant, Ser742Tyr, demonstrated stronger binding to the *BACE1*-encoded enzyme than the wild-type protein, raising the possibility that altered filaggrin–BACE1 interactions could modulate amyloidogenic processing. Bioinformatics analyses further linked *FLG* to pathways involved in nervous system development and axonal regeneration, lending additional plausibility to its role as a cross-disease mediator. While these findings remain preliminary and do not establish a direct genetic association between atopic dermatitis and AD, they nominate *FLG* as a potential biological bridge between skin-barrier dysfunction and neurodegeneration [70].

This study has several notable strengths. By applying a rare-variant association framework to the ADNI cohort, the authors moved beyond conventional GWAS approaches and identified *FLG* as a novel candidate gene in AD. The detection of a classical epidermal-barrier gene in the context of dementia represents an original and provocative finding. Importantly, the study complemented statistical evidence with mechanistic exploration, using molecular docking and dynamics simulations to show that filaggrin interacts with the *BACE1*-encoded β-site APP-cleaving enzyme 1, the central enzyme in amyloid-β generation. Pathway enrichment further linked *FLG* to nervous-system development and axonal regeneration, providing additional biological plausibility [70].

However, several limitations must be considered. The analysis did not directly test whether atopic dermatitis and AD co-occur genetically, but instead focused on *FLG* as an overlapping locus. The findings rely on computational predictions and statistical associations, without functional validation in cellular or animal models. The study cohort, drawn from ADNI, is relatively small and largely European, which restricts power and generalizability across populations. Furthermore, much of the mechanistic inference centered on a single variant (*Ser742Tyr*), and it remains unclear whether other *FLG* variants exert similar effects. Thus, while the work highlights *FLG* as a potential biological bridge between barrier dysfunction and neurodegeneration, replication in larger, multi-ethnic cohorts and experimental validation will be essential to clarify its role [70].

Exploring the potential link between atopic eczema and dementia is of clinical importance, as it could enable earlier identification of individuals at elevated risk for cognitive decline and inform strategies to reduce long-term disease burden. In conclusion, converging epidemiological and genetic data support a possible link between atopic dermatitis and dementia. While epidemiological studies point to an elevated risk of cognitive decline in patients with eczema, emerging rare variant analyses nominate *FLG* as a potential genetic mediator connecting skin barrier dysfunction to AD biology. These findings remain preliminary but raise important questions about shared mechanisms that could, in the future, inform risk stratification and novel therapeutic strategies.

#### 3.2.5. Possible Disease-Specific Mechanistic Axis Linking Atopic Dermatitis and Dementia

Integrating epidemiological, genetic, and mechanistic evidence, the connection between atopic dermatitis and dementia appears to involve immune-inflammatory, vascular, and barrier-gene pathways rather than direct inherited causality. Chronic Th2-skewed inflammation in atopic dermatitis, characterized by sustained IL-4, IL-13, and IL-6 signaling, can disrupt the BBB, promote microglial activation, and induce neuroinflammatory cascades that impair Aβ clearance. Endothelial activation and oxidative stress further compromise cerebral perfusion, fostering neurovascular dysfunction [69]. At the molecular level, rare-variant bioinformatics analysis identified *FLG*, with variant-induced strengthening of filaggrin–*BACE1* interactions that may subtly promote amyloidogenic processing [70]. Although Mendelian randomization studies [69] do not confirm a causal genetic overlap, these emerging data suggest that skin-barrier genes, chronic systemic inflammation, and cerebrovascular stress may intersect along a shared biological continuum, the skin–brain axis, linking cutaneous barrier failure with neurodegenerative vulnerability.

#### 3.2.6. Genetic and Biological Evidence Linking Rosacea and Atopic Dermatitis to Dementia

Viewed together, the current evidence for rosacea and atopic dermatitis highlights a continuum of support for a biological rather than a strictly genetic bridge with dementia. Rosacea exhibits strong proteomic and transcriptomic overlap with neurodegenerative pathways; however, no validated germline associations have been established to date. Instead, several shared regulatory hubs have been identified, converging on signaling axes. In contrast, atopic dermatitis introduces preliminary genetic insights through rare *FLG* variants, suggesting a potential mechanistic connection to amyloidogenic processing and thus a more direct interface with Alzheimer’s disease biology.

These findings are summarized in Table 3, which outlines the type of evidence, key molecular players, and the qualitative strength of support across inflammatory skin diseases studied in relation to dementia.

### 3.3. Bullous Pemphigoid and Emerging Neurogenic Link with Dementia

#### 3.3.1. Epidemiology and Clinical Evidence

Bullous pemphigoid (BP), an autoimmune blistering disease predominantly affecting the elderly, has been increasingly linked to neurodegenerative disorders, particularly Alzheimer’s and Parkinson’s disease. Epidemiologic studies demonstrate that individuals with BP have a significantly higher risk of neurological comorbidities, including dementia, which frequently precedes the onset of cutaneous disease. A large Taiwanese population-based cohort found that 17.7% of BP patients had a prior diagnosis of dementia compared with 4.6% of controls, corresponding to an adjusted odds ratio of 3.04 (95% CI 2.67–3.46) [71]. Similarly, a 50-year population study from Olmsted County, Minnesota, reported that 10% of BP patients had dementia at the time of diagnosis versus 2% of controls, yielding an odds ratio of 6.75 (95% CI 2.08–21.92), which increased to 9.00 (95% CI 2.44–33.24) in those with generalized BP [72]. In both studies, dementia typically preceded BP by several years, suggesting that neurodegenerative processes may initiate or enhance autoimmune reactivity through antigen exposure and immune cross-reactivity, ultimately linking neurological degeneration with subsequent blistering skin disease.

#### 3.3.2. Shared Autoantigens (BP180, BP230)

Mechanistically, BP autoantigens, BP180 (collagen XVII, also known as BPAG2) and BP230, are not restricted to the skin but are also expressed in neurons of several brain regions, including the cerebral cortex, hippocampus, basal nuclei, and substantia nigra. Neurological diseases may lead to neuronal injury and compromise of the BBB, exposing these normally sequestered antigens to the immune system and triggering an autoimmune response that subsequently manifests as cutaneous disease. Supporting this neurocutaneous model, antibodies against collagen XVII have been detected in the serum of patients with dementia and other neurological disorders, and sera from BP patients with neurological comorbidities recognize both BP180 and BP230 in human brain extracts. These findings strengthen the hypothesis that collagen XVII serves as a shared antigen bridging the central nervous system and skin, supporting the epidemiological association between BP and neurodegenerative disease [73].

#### 3.3.3. Immunogenetic Susceptibility (HLA-DQB1*03:01)

To date, very limited studies have investigated the genetic connections between BP and neurodegeneration. Amber et al. [74] proposed a multi-hit hypothesis to explain the frequent co-occurrence of BP with neurological disease, focusing on the immunogenetic role of the HLA-DQB1*03:01 allele. Drawing on prior immunogenetic, histopathologic, and neuroimmunological data, the authors suggested that this allele enhances T-cell binding affinity for epitopes within BP180, particularly the NC16a domain, predisposing carriers to loss of tolerance once neuronal injury exposes normally sequestered antigens. This model elegantly integrates the well-documented neuronal expression of BP180 and BP230 with epidemiologic observations showing that neurological disease, most commonly dementia, PD, or stroke, precedes BP onset in approximately 70% of cases, often by several years. The findings, supported by earlier evidence that these autoantigens are expressed in neurons of the cortex, hippocampus, and substantia nigra and that sera from patients with BP or neurodegenerative disease can recognize brain-derived BP180, reinforce a neurocutaneous immune axis whereby neurodegeneration may act as the initiating event in cutaneous autoimmunity [74].

A key strength of Amber et al.’s [74] work lies in its conceptual synthesis of molecular immunology, neurobiology, and clinical epidemiology, providing a coherent mechanistic framework that explains both the directionality and antigenic specificity of the BP–neurodegeneration association. However, the hypothesis remains theoretical and indirect, derived largely from immunogenetic correlations, in silico modeling, and extrapolation from animal studies rather than direct functional or longitudinal patient data. The absence of prospective genetic validation and the limited assessment of non-HLA immune or environmental modifiers constrain causal inference. Nevertheless, the study represents a significant conceptual advance, uniting dermatologic and neurologic autoimmunity under a shared antigen hypothesis and generating testable predictions for future multi-omic and translational research.

### 3.4. Hidradenitis Suppurativa and the γ-Secretase Pathway

Hidradenitis suppurativa (HS, acne inversa) is a chronic, relapsing inflammatory skin disease affecting approximately 1% of adults and characterized by painful nodules, abscesses, and sinus tracts in apocrine-gland–bearing areas [75]. Beyond its cutaneous manifestations, HS has been examined for potential links to neurodegenerative disorders, particularly AD, owing to shared alterations in the γ-secretase complex, a transmembrane protease responsible for the intramembranous cleavage of multiple substrates, including Notch and amyloid precursor protein (APP) [76].

Familial HS most commonly involves heterozygous loss-of-function variants in γ-secretase subunit genes such as *NCSTN* (nicastrin), *PSENEN* (presenilin enhancer 2), and *PSEN1* (presenilin-1) [77]. These truncating or frameshift mutations reduce γ-secretase activity and impair Notch signaling, leading to follicular occlusion, epidermal hyperplasia, and chronic inflammation. In contrast, autosomal-dominant familial AD arises primarily from gain-of-function missense or in-frame mutations in *PSEN1* or *PSEN2*, which alter γ-secretase cleavage of APP and increase production of the neurotoxic Aβ42 peptide [78]. Thus, although HS and AD converge mechanistically on γ-secretase dysfunction, their molecular outcomes diverge: reduced enzymatic activity underlies HS pathogenesis, whereas aberrant APP processing promotes amyloid deposition and neurodegeneration in AD. Together, these findings highlight γ-secretase as a shared mechanistic node linking epithelial and neuronal homeostasis through distinct modes of dysregulation rather than a single causal genetic bridge [79].

This mechanistic overlap was further explored at the genetic level in the only human study, to the best of our knowledge, to date, investigating a possible connection between HS and AD. In a genetic linkage and sequencing analysis of six unrelated Han Chinese families with autosomal-dominant HS, Wang et al. [76] identified heterozygous loss-of-function variants in *PSENEN*, *NCSTN*, and *PSEN1* [76]. Because *PSEN1* and *PSEN2* mutations also underlie early-onset familial AD, these findings suggest a shared molecular pathway linking cutaneous and neurodegenerative biology through disrupted γ-secretase activity. However, the overlap appears mechanistic rather than allelic since AD-associated *PSEN1* mutations are typically missense or in-frame substitutions that alter substrate processing, whereas HS-associated variants are truncating or frameshift mutations leading to haploinsufficiency and loss of function. Clinically, none of the 50 affected individuals studied, including 15 over the age of 50, showed evidence of dementia or cognitive decline, although a comprehensive neurological evaluation was not performed. Consequently, while HS and AD both implicate γ-secretase dysfunction, current data do not support a direct genetic overlap. Major limitations include the small familial sample size, lack of longitudinal neurocognitive assessment, and absence of functional validation to determine whether HS-linked *PSEN1* mutations exert amyloidogenic effects in neural tissue [76].

Epidemiological data examining the HS and AD relationship remain inconclusive. A large retrospective population-based cohort study conducted in 2017 compiles longitudinal clinical data from 27 U.S. health systems and approximately 48 million unique patients, identified 28,755 individuals with HS [80]. Diagnoses of HS and AD were obtained using standardized clinical coding, and analyses were adjusted for age and sex. The absolute risk of AD among patients with HS was 0.2% (65 of 28,755). While crude analysis suggested a lower AD risk in HS (odds ratio [OR] = 0.34; 95% CI 0.26–0.43), adjusted analysis rendered the association modest and statistically nonsignificant (adjusted OR = 1.23; 95% CI 0.96–1.56). These findings were consistent with prior Danish registry data [81] reporting no significant relationship between the two conditions. The authors concluded that, despite overlapping γ-secretase biology, HS does not appear to confer an increased epidemiologic risk of AD, though their analysis could not account for genetically defined familial HS subgroups carrying *PSEN* or *NCSTN* mutations—an important limitation [80].

In contrast, a later Turkish cross-sectional study involving 192 HS patients [82] assessed family history of both HS and AD. None of the participants had AD themselves, but 9.4% reported a family history of the disorder. The prevalence of AD family history was significantly higher among patients with a family history of HS (25.8%) compared with those without (6.2%), corresponding to a 4.5-fold increased risk; this rose to an 8.8-fold increase among patients aged 40 years or older. The authors contrasted their findings with earlier large-scale cohort studies, suggesting that the absence of an observed association in those investigations may reflect the limitations of retrospective, code-based analyses with short follow-up durations, which could miss familial or long-term trends [82].

Overall, while γ-secretase biology provides a plausible molecular intersection between HS and AD, current evidence does not support a consistent or causal genetic link. Future prospective, longitudinal, and genotype-stratified studies are warranted to clarify how specific γ-secretase variants influence disease expression and whether they modulate risk for neurodegenerative comorbidity. Such investigations could also elucidate how genetic, environmental, and hormonal factors interact over time, ultimately advancing a more comprehensive understanding of HS pathogenesis and informing personalized therapeutic strategies [83].

### 3.5. Seborrheic Dermatitis, Vitiligo, and Prurigo Nodularis

A recent review addressing the relationship between skin inflammation and dementia also discusses several less-studied inflammatory dermatoses, such as seborrheic dermatitis, prurigo nodularis, and vitiligo, which may be associated with cognitive dysfunction and neurodegenerative disease. These conditions share systemic inflammatory characteristics, barrier impairment, and immune dysregulation, factors increasingly recognized as relevant to neuroinflammation and neuronal decline [6]. For example, in a case report study from Italy, patients with prurigo nodularis have been reported to exhibit varying degrees of cognitive impairment, ranging from psychiatric symptoms and mild cognitive dysfunction to AD [84]. Similarly, vitiligo, an inflammatory skin disorder marked by melanocyte loss and impaired epidermal barrier recovery, has been linked to a higher incidence of dementia (5.0 vs. 1.0 per 1000 person–years; adjusted HR = 5.3), although long-term follow-up data suggest that the risk of AD may not differ significantly from controls [85,86]. Evidence on seborrheic dermatitis remains limited but points toward a possible association with systemic comorbidities and neurodegenerative changes [6].

At present, there is no confirmed genetic association between these inflammatory skin diseases and dementia. The observed relationships likely reflect immune-mediated, inflammatory, and metabolic mechanisms rather than shared heritable susceptibility. Nonetheless, the inclusion of these disorders is important from a holistic perspective, as they accentuate the potential influence of chronic peripheral inflammation on central nervous system health. Future studies integrating immunogenetic, transcriptomic, and clinical data may help elucidate how inflammatory skin processes contribute to or mirror neurodegenerative pathways.

## 4. Huntington’s Disease and the Peripheral Mirror of Neurodegeneration

HD is a genetic neurodegenerative disorder caused by an expanded CAG trinucleotide repeat in the *HTT* gene, leading to production of mutant huntingtin protein (mHTT). This mutation induces mitochondrial dysfunction and disrupts the ubiquitin–proteasome system in neurons, resulting in progressive motor, cognitive, and psychiatric impairment. Although rare, HD is a globally distributed neurodegenerative disorder, with prevalence approaching 5 per 100,000 worldwide, highest in populations of European ancestry, and stable to slightly increasing rates over recent decades, largely due to diagnostic and survival improvements rather than a true rise in new cases [87].

Peripheral manifestations of HD have been increasingly recognized with recent transcriptomic analyses identifying significant upregulation of *PLCB4*, *UBE2D3*, *APC*, and *ROCK1* in fibroblasts derived from HD patients compared with controls. These genes are involved in RNA processing, cellular signaling, and cytoskeletal organization, mirroring molecular disruptions seen in neuronal tissue [88]. Moreover, increased expression of parkin protein in juvenile HD fibroblasts appears to exert a protective role by enhancing proteasome activity and maintaining mitochondrial integrity [89]. Together, these findings highlight the potential of skin fibroblasts as accessible peripheral models for exploring systemic molecular alterations in HD and for identifying novel diagnostic and therapeutic targets; however, to the best of our knowledge, there are no current genetic studies directly associating inflammatory skin diseases with HD [50].

Although these findings do not implicate inflammatory skin diseases directly in HD genetics, they provide compelling evidence that the skin reflects systemic molecular changes associated with neurodegeneration. Given this overlap, the skin serves as a valuable peripheral model for studying HD and may, in the future, reveal novel genetic or inflammatory interactions bridging cutaneous and neural pathology. Therefore, it is important to acknowledge this emerging skin–brain connection within the context of neurogenetic research, as it may guide future studies toward identifying shared biomarkers and therapeutic targets [50].

## 5. Integrative Perspective

**Figure 1:**
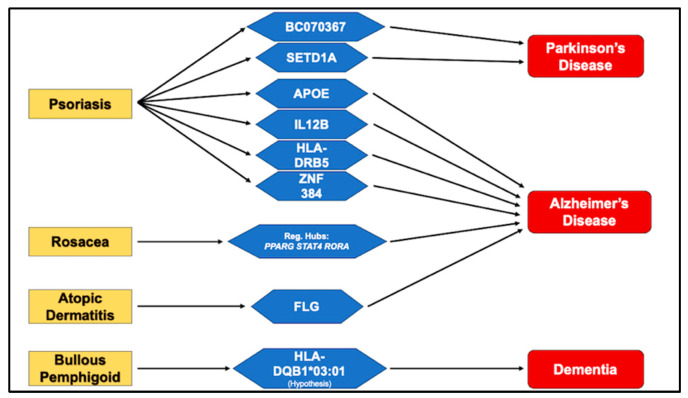
Genes Bridging Cutaneous Inflammation and Cognitive Decline.

As schematically illustrated in Figure 1, this review consolidates current evidence implicating genetic mediators at the interface of cutaneous inflammation and dementia, revealing both convergent mechanisms and unresolved controversies. Converging genetic, immunologic, and transcriptomic evidence supports the existence of a shared skin–brain axis connecting inflammatory dermatoses and neurodegenerative disorders. Among these disease pairings, psoriasis and AD currently represent the most substantiated model. Susceptibility at *APOE*, *IL12B*, and variants near *HLA-DRB5*, together with transcriptomic regulators such as *ZNF384*, delineate a molecular bridge linking cutaneous inflammation with neurodegenerative vulnerability through dysregulated IL-17, IL-23, and TNF signaling and impaired antigen presentation [27,38,40,43,44].

Complementary pleiotropy-informed analyses have identified additional loci, including *SETD1A* and *BC070367*, connecting psoriasis and Parkinson’s disease via immune–epigenetic interactions [55]. Clinically, ongoing research by Levitt et al. [35] demonstrated that psoriasis patients treated with biologic agents targeting IL-17, IL-23, IL-12/23, or TNF-α exhibited a nearly 50% reduction in dementia incidence compared with those receiving conventional systemic therapy, suggesting that sustained cytokine inhibition may mitigate systemic and neuroinflammatory cascades contributing to cognitive decline [35].

Beyond psoriasis, other chronic inflammatory dermatoses illustrate distinct yet convergent mechanisms along this axis. In rosacea, proteomic and transcriptomic investigations have revealed enrichment of neurodegeneration-related proteins such as *SNCA, GSK3B*, and *HSPA8,* as well as regulatory hubs including *PPARG*, *STAT4*, and *RORA*, which converge on NF-κB, IL-17, and TNF signaling pathways [62,64]. These findings indicate that persistent cutaneous inflammation and vascular dysregulation may recapitulate early neurodegenerative processes. In atopic dermatitis, rare-variant analyses have highlighted *FLG* as a potential cross-disease gene [70]. Computational modelling suggests that the *FLG* Ser742Tyr variant enhances binding to *BACE1*, the β-site APP-cleaving enzyme critical for amyloid-β generation, supporting a barrier–amyloid axis linking epidermal dysfunction to AD [70]. BP represents a complementary autoimmune model, where shared neuronal and epidermal expression of *BP180* (collagen XVII) and *BP230*, together with the HLA-DQB1*03:01 allele, support a neurocutaneous autoimmunity hypothesis [73,74]. Epidemiological data consistently show that dementia often precedes the onset of bullous pemphigoid, implying that neurodegeneration may expose normally sequestered neuronal antigens, thereby triggering secondary autoimmune responses in the skin [71,72].

Geographic and ethnic epidemiological differences add further complexity, as both inflammatory dermatoses and neurodegenerative diseases exhibit variable prevalence and severity across populations. Gradients in allele frequencies, such as the north–south distribution of *APOE* ε4 or regional variation in *IL12B* polymorphisms imply that heritable factors contribute to these regional disparities [14,30,31]. Such findings strengthen the argument for genetic involvement in the observed comorbidity patterns and highlight the importance of multi-ethnic, ancestry-aware genetic studies in understanding the architecture of the skin–brain axis.

Despite increasing clarity, substantial knowledge gaps remain. In BP, the biological and molecular interactions with neurodegeneration remain speculative, grounded primarily in immunogenetic and serologic observations [73,74]. Similarly, HS epidemiological associations with dementia and AD remain inconclusive, and mechanistic and genomic data are inadequate [73,74]. Conversely, HD, a neurodegenerative disease, provides an inverse example. It is closely associated with skin fibroblast dysfunction, as these cells recapitulate neurodegenerative hallmarks such as mitochondrial stress and defective autophagy, yet no evidence currently links HD to inflammatory dermatoses [88,89].

This illustrates that while the skin may mirror neural degeneration at the cellular level, inflammatory and genetic pathways do not universally overlap across all neurodegenerative disorders.

## 6. Strengths and Limitations

The body of current evidence exhibits several methodological strengths. Integrative multi-omic approaches combining genomic, transcriptomic, and proteomic analyses have revealed convergent immune and metabolic pathways across both systems, reinforcing biological plausibility. Large-scale, population-based cohorts from Korea, Germany, and Israel have strengthened epidemiologic validity, while the alignment between genetic discoveries, such as *IL12B*, and the therapeutic efficacy of corresponding biologics, such as ustekinumab, provides translational coherence linking genotype, cytokine modulation, and clinical outcomes [14,30,35,39,52].

Nevertheless, important limitations constrain interpretation. Most studies remain cross-sectional or retrospective, relying on registry-based diagnoses that may introduce misclassification bias. Genetic analyses have primarily focused on European and East Asian populations, restricting global generalizability. The strength of association for rarer dermatoses such as BP and rosacea is limited by small sample sizes, while findings for atopic dermatitis are based largely on computational models without experimental validation. Furthermore, causality is often inferred indirectly through statistical pleiotropy or expression overlap rather than demonstrated through mechanistic studies. The absence of longitudinal cognitive, neuroimaging, or biomarker endpoints also limits the ability to determine whether these associations translate into true neuroprotective benefit. Environmental and lifestyle modifiers, including microbiome composition, diet, and pharmacologic exposure, remain underexplored within genetic frameworks, further complicating causal interpretation.

Future research should focus on addressing these gaps through multi-ethnic, longitudinal, and mechanistic studies that integrate genomic, transcriptomic, and metabolomic data. Large international consortia linking dermatologic and neurologic biobanks could enable cross-phenotype genome-wide and rare-variant analyses to dissect shared and population-specific risk. Functional studies using induced pluripotent stem cell-derived keratinocytes, glia, and neurons may clarify how disease-associated variants alter cytokine networks, barrier integrity, and neuroimmune interactions. In parallel, clinical trials of biologic and systemic immunomodulators should incorporate neurocognitive and neuroimaging endpoints to determine whether early, sustained control of systemic inflammation can attenuate neurodegenerative progression.

## 7. Future Directions

From a clinical perspective, the emerging convergence of evidence argues for the introduction of routine MCI screening in patients with chronic inflammatory dermatoses. More broadly, recognizing inflammatory skin diseases, sex and neurodegenerative disorders as interconnected systemic processes rather than isolated organ-specific conditions may redefine future prevention and treatment paradigms. Early recognition of cognitive decline would facilitate timely therapeutic intervention, enable individualized patient management, and improve long-term quality of life. Furthermore, future studies should explore the potential neuroprotective effects of biologic and anti-inflammatory agents used in skin inflammatory diseases, currently psoriasis, as emerging evidence suggests that modulation of systemic inflammation through IL-17, IL-23, or TNF-α inhibition may attenuate neuroinflammatory pathways and reduce the risk or progression of neurodegeneration.

In summary, the cumulative data suggest that the skin–brain axis is governed by a shared immunogenetic circuitry involving cytokine signaling, lipid metabolism, antigen presentation, and neuroimmune cross-talk. Geographic variation, differential genetic predisposition, sex and environmental influences all contribute to the heterogeneity observed across populations. Bridging these findings through integrative, multi-ethnic, and mechanistic research holds the potential to establish a new precision medicine framework at the interface of dermatology and neurology, one that not only controls cutaneous inflammation but also preserves cognitive health and extends the quality and longevity of life for affected individuals.

## 8. Conclusions

Taken together, these insights redefine inflammatory skin diseases as systemic disorders with neurological relevance rather than purely cutaneous entities. The recognition of shared genetic and immunologic determinants between the skin and brain not only reshapes pathophysiological understanding but also opens translational opportunities for early neurocognitive screening and cytokine-targeted intervention. Integrating dermatology and neurology through precision immunogenetics could transform patient care, enabling the prevention of neurodegeneration through the timely control of systemic inflammation. Ultimately, elucidating the molecular dialogue between the epidermis and cortex may allow clinicians to treat the skin as both a diagnostic window and a therapeutic gateway to the brain.

## Figures and Tables

**Table 1:** Shared genetic mediators linking psoriasis and AD.

Gene/Locus	Evidence in Psoriasis	Evidence in AD	Strength of Evidence	Source
*APOE* (ε2, ε3, ε4 alleles)	Candidate-gene studies and meta-analyses show ε2 overrepresented, ε3 protective, ε4 linked to severity in some cohorts.	ε4 allele is the strongest and most replicated genetic determinant of late-onset AD; ε2 protective, ε3 neutral.	True genetic association in both diseases; robust in AD, modest/inconsistent in psoriasis.	[24,27]
*IL12B* (rs3212227, rs6887695)	Strongly validated psoriasis locus; replicated across cohorts; biologic therapies against IL-12/23p40 confirm functional role.	Case–control study in Han Chinese shows rs3212227 associated with AD risk reduction; not consistently replicated in European cohorts.	Established genetic association in psoriasis; preliminary and population-specific in AD.	[38,40]
*HLA* region (rs2516049 near HLA-DRB5)	GWAS identifies a variant as a susceptibility locus for psoriasis.	Same variant linked to AD risk, neurofibrillary tangle burden, and cognitive decline.	True GWAS-level genetic overlap; causal variant unclear due to HLA locus complexity.	[43]
*ZNF384* (transcriptional regulator)	Differentially expressed in psoriatic lesions; predicted to regulate multiple inflammatory/metabolic genes.	Dysregulated in AD hippocampus; implicated in JAK–STAT and metabolic imbalance.	Shared transcriptomic/regulatory signature, not a proven genetic risk locus.	[44]
Other transcription factors (*PPARG, ZFPM2, ZNF415, HLX, ANHX*)	Identified in psoriasis transcriptomic networks.	Similarly dysregulated in AD brain tissue.	Convergent expression/regulatory findings; no direct genetic association.	[44]

**Table 2:** Genetic loci with pleiotropic associations in psoriasis and PD.

Locus/ Variant	Effect in Psoriasis	Effect in Parkinson’s Disease	Interpretation	Source
*SETD1A* (rs11640961)	Increased risk	Reduced risk	Supported by pleiotropy-based GWAS and replication. The association is moderately robust, but opposite effects suggest divergent or context-dependent biological roles.	[55]
*BC070367* (rs1975974)	Increased risk	Increased risk	Identified through pleiotropy analysis with concordant effects. The signal is weaker and less replicated, and its functional relevance remains uncertain.	[55]

**Table 3:** Biological and genetic evidence linking inflammatory skin diseases to dementia.

Disease	Evidence Type	Key Genes/Proteins/Regulatory Hubs	Mechanistic Implication	Strength of Evidence	Source
Rosacea	Proteomic/Transcriptomic/Regulatory Network	*SNCA, GSK3B, HSPA8;* Regulatory hubs: *PPARG*, *STAT4*, *RORA*; NF-κB/IL-17/TNF axis	Shared inflammatory, neurovascular, and proteostatic pathways; transcriptional convergence on NF-κB, IL-17, and TNF signaling	Biological only. No validated germline association	[62,64]
Atopic Dermatitis	Rare-variant/Genetic/Computational Modeling	*FLG* (Ser742Tyr); filaggrin–*BACE1* interaction	Barrier dysfunction, rare-variant-driven modulation of amyloidogenic processing, neurovascular stress	Preliminary genetic. Requires replication and functional validation	[70]

## Data Availability

No new data were created or analyzed in this study. Data sharing is not applicable to this article.

## Abbreviations

Alzheimer’s Disease (AD), Late-Onset AD (LOAD), Parkinson’s Disease (PD), Machine Learning (ML), Mild Cognitive Impairment (MCI) Central Nervous System (CNS), Blood–Brain Barrier (BBB), Alzheimer’s Disease Neuroimaging Initiative (ADNI) Hazard Ratio (HR), *Amyloid-β* (*Aβ*) Genome-Wide Association Study (GWAS), Differentially Expressed Genes (DEGs), Matrix Metalloproteinases (MMPs), α-synuclein (SNCA) Adenosine Monophosphate (AMP), *Filaggrin* (*FLG*), Bullous pemphigoid (BP), Hidradenitis Suppurativa (HS), Amyloid Precursor Protein (APP).
